# Advances in understanding the regulation of pluripotency fate transition in embryonic stem cells

**DOI:** 10.3389/fcell.2024.1494398

**Published:** 2024-10-16

**Authors:** Yong kang Jia, Yang Yu, Li Guan

**Affiliations:** ^1^ School of Life and Health Sciences, Hubei University of Technology, Wuhan, China; ^2^ Guangzhou Women and Children’s Medical Center, Guangzhou Medical University, Guangzhou, China

**Keywords:** ESCs, 2-cell-like cells, Dux, MERVL, epigenetic modification, cell fate reprogramming

## Abstract

Embryonic stem cells (ESCs) sourced from the inner cell mass of blastocysts, are akin to this tissue in function but lack the capacity to form all extraembryonic structures. mESCs are transient cell populations that express high levels of transcripts characteristic of 2-cell (2C) embryos and are identified as “2-cell-like cells” (2CLCs). Previous studies have shown that 2CLCs can contribute to both embryonic and extraembryonic tissues upon reintroduction into early embryos. Approximately 1% of mESCs dynamically transition from pluripotent mESCs into 2CLCs. Nevertheless, the scarcity of mammalian embryos presents a significant challenge to the molecular characterization of totipotent cells. To date, Previous studies have explored various methods for reprogramming pluripotent cells into totipotent cells. While there is a good understanding of the molecular regulatory network maintaining ES pluripotency, the process by which pluripotent ESCs reprogram into totipotent cells and the associated molecular mechanisms of totipotent regulation remain poorly understood. This review synthesizes recent insights into the regulatory pathways of ESC reprogramming into 2CLC, exploring molecular mechanisms modulated by transcriptional regulators, small molecules, and epigenetic changes. The objective is to construct a theoretical framework for the field of researchers.

## Introduction

Totipotent stem cells typically refer to fertilized eggs and blastomeres of 2-cell embryos formed following fertilization. During this stage, there is activation of the zygotic genome accompanied by epigenetic reprogramming, establishing their ability to give rise to both the embryo and all extraembryonic tissues (Lee et al., 2014; Genet and Torres-Padilla, 2020). As development advances to the blastocyst stage, these totipotent cells differentiate into pluripotent embryonic stem cells (ESCs), which possess the ability to differentiate into cell types originating from all three germ layers within the inner cell mass (ICM). ESCs serve as an invaluable *in vitro* model for studying early embryo characteristics during the preimplantation stage. Despite the uniform capacity for self-renewal among ESCs, in 2012, Todd S. Macfarlan and colleagues identified a heterogeneous population within mouse ESC (mESC) cultures, exhibiting distinct functional attributes. This subset of cells displays a unique transcript expression and active histone modifications reminiscent of blastomeres from 2-cell stage embryos. Approximately 1% of this population, designated as 2-cell-like cells (2CLCs), exhibit gene expression and epigenetic profiles analogous to those of early embryos (Macfarlan et al., 2012; Zhu et al., 2021b). This discovery has facilitated the establishing *in vitro* models of resembling totipotent cells, crucial for investigating cell lineage development and advancing regenerative medicine.

For decades, researchers have employed embryonic stem cells (ESCs) as a model to investigate strategies for reprogramming them into a 2-cell-like state. However, to date, no definitive culture system has been established (Fu et al., 2020). The primary challenge lies in the inherent instability of the 2CLC state; its totipotent nature is transient, with most 2CLC spontaneously reverting to their original pluripotent state during *in vitro* culture. The complete process involving the emergence and regression of the spontaneous 2CLC state, along with its associated molecular mechanisms, remains incompletely understood (Zhu et al., 2021a). Another obstacle stems from the distinction between mESCs obtained through genetic modification or cultivated under different regulatory conditions, and the 2-cell stage embryonic cleavage spheres at the transcriptional level. This discrepancy suggests a contentious issue regarding their developmental potential (Posfai et al., 2021). Consequently, experimental inquiries into totipotency confront substantial challenges, and the establishment of authentic totipotent embryonic stem cells has not been achieved. Recent research has underscored the role of diverse factors, including transcriptional factors, non-coding RNAs, small chemical molecules, and epigenetic modifications, in either promoting or impeding the transition to the 2CLC state. In this review, we aim to elucidate the cumulative progress regarding the diverse molecular mechanisms governing the transition from pluripotency to totipotency in mammalian embryonic stem cells. Our objective is to provide insights to researchers in related fields and offer novel perspectives for the application of stem cell regenerative medicine.

### The molecular characteristics of 2-cell-like cells (2CLC)

2C-like cells were initially identified in experiments conducted by Pfaff’s Lab, where ESC was labeled with the repetitive element “MERVL-L-Gag” (Macfarlan et al., 2011). These repetitive elements are predominantly active during the late 2-cell embryo stage of mouse zygote genome activation (ZGA), with transcription of mouse endogenous retroviruses reaching a peak at this stage. During the 2-cell embryo stage, coinciding with the initial activation of the zygote genome, the transcription of MERVL family retroviral genetic elements is highly transcribed, subsequently undergoing rapid attenuation as development advances. Consequently, the approach employing the transcription of MERVL family retroviral genetic elements to induce the expression of fluorescent genes as a reporting system via their long terminal repeats (LTRs) offers promising avenues for the genetic labeling of 2C-like cells *in vitro*. This sheds light on critical developmental processes during this embryonic phase. In addition to the elevated expression of MERVL transcripts, 2CLCs demonstrate enhanced reprogramming efficacy towards a variety of cell fates. Pluripotency factors essential for preserving ESC pluripotency and their capacity for self-renewal, including OCT4, SOX2, and NANOG, are initially expressed at low levels and increase gradually during development (Komatsu and Fujimori, 2015). 2C-like cells (2CLC) are distinguished by specific high-level transcripts from several gene families, which serve as valuable markers for their identification and characterization. Key markers include:

Zfp352: Associated with pluripotency and reprogramming (Mwalilino et al., 2023).
MERVL-int/MT2-Mm: Transposons involved in gene expression regulation and stem cell maintenance (Sakashita et al., 2023).
Eif1a-like cluster: Genes related to translation initiation and protein synthesis, potentially impacting reprogramming efficiency (Hung et al., 2013).
Zscan4 cluster: Genes critical for genome stability and repair (Zalzman et al., 2010).

The unique molecular signature, characterized by the robust expression of these gene families, distinctly delineates 2CLCs. This distinctive expression profile not only constitutes a definitive marker for 2C-like cells but also plays a pivotal role in their reprogramming efficacy and the potential to specify cell destiny.

Subsequent phenotypic examination of 2CLCs uncovered attributes akin to those observed in 2-cell stage embryos. This includes de-condensation of chromocenters (Ishiuchi et al., 2015), enhanced nucleosome mobility (Bošković et al., 2014; Ooga et al., 2016), chromatin remodeling (Jansz and Torres-Padilla, 2019; Kruse et al., 2019), reduced DNA methylation (Eckersley-Maslin et al., 2016), and increased levels of transcriptionally activating histone modifications (Dahl et al., 2016; Rodriguez-Terrones et al., 2020). In addition to their transcriptional and chromatin-related characteristics, 2CLCs exhibit unique metabolic attributes, including diminished glycolytic activity, reduced mitochondrial respiratory capacity, and a lower rate of oxygen consumption relative to ESCs(Kaneko, 2016). In conclusion, 2CLCs constitute a transient population of pluripotent cells that harbors specific molecular, chromatin, nuclear organizational, and metabolic characteristics akin to those of 2-cell stage embryo blastomeres. The detailed characterization underscores the importance of 2CLCs as a valuable cellular model for investigating early embryonic development and the biology of pluripotent cells.

## The regulatory mechanism of ES cells reprogramming into 2-cell-like cells

The fundamental nature of cell fate transitions is primarily driven by extensive reprogramming of epigenetic information. The spontaneous conversion of ES cells to 2-cell embryo-like cells can occur *in vitro* under culture conditions that include serum and leukemia inhibitory factor (LIF). Research indicates that this reprogramming process involves a complex regulatory network, featuring key genetic elements such as the Zscan4 gene, endogenous retrovirus MERVL, transcription factor Dux, epigenetic modifiers, and signaling pathway-associated proteins. Moreover, the potential to induce ES cells into 2C-like cells using small chemical molecules opens promising avenues for precise modulation of cell fate.

### Transcriptional regulation of Dux mediates reprogramming of ES cells into 2C-like cells

The mouse double homeobox gene (Dux), also known as DuxF3, and its human counterpart, double homeobox 4 (Dux4), encode a double homeodomain transcription factor that is crucial for driving pluripotency. These genes are specifically expressed during the ZGA stage of early embryonic development, particularly at the 2-cell stage. In ESCs, mouse DUX can activate the ERVL family of repetitive sequences and ERVL-linked genes, playing a critical role in the transition from ESCs to 2CLC. However, DUX alone is not entirely sufficient for this transition. Transcriptome analysis reveals that the genomic sequences of Dux and Dux4 are typically located in heterochromatin regions and remain silent in most cells, including ESCs. Their expression is activated during the ZGA stage and during the reprogramming of ESCs to 2CLC. The precise activation of these genes is tightly regulated by multiple mechanisms to ensure proper developmental progression.

### Epigenetic modification regulates Dux activation to control the fate transformation of 2C-like cells

**FIGURE 1:**
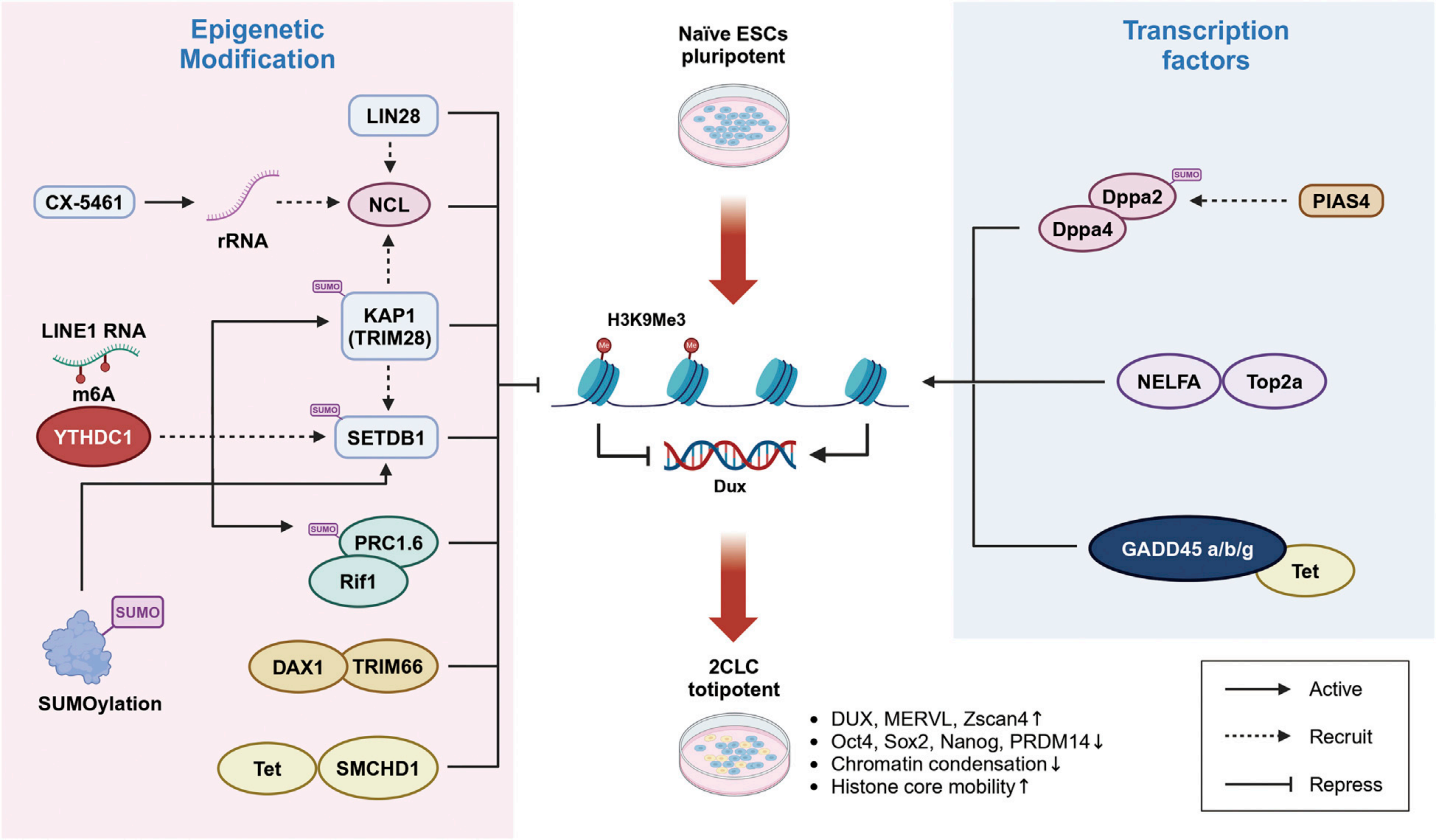
Transcriptional regulation of Dux in reprogramming of ES cells cells into 2C-like cells. Left: The regulation of Dux expression involves several epigenetic modifications. Increased levels of H3K9Me3 modification, mediated by the activation of the histone H3 methyltransferase SETDB1 through YTHDC1, NCL, and LIN28, contribute to the silencing of Dux expression. Additionally, epigenetic regulators such as TRIM66 and SMCHD1 also play a role in suppressing Dux. Right: The activation of Dux expression is regulated by transcription factors. Dppa2/4 and NELFA bind to the Dux promoter region, facilitating its activation. Furthermore, DNA demethylation mediated by GADD45a/b/g is essential for activating Dux expression.

The expression of genes is primarily regulated by epigenetic modifications. Epigenomic analyses reveal a correlation between increased levels of open chromatin histone modifications, such as H3K27ac, H3K4me1, and H3K4me3, and the transition process to 2C-like cells (Zhang et al., 2021; Wu et al., 2023). These modifications are associated with Dux binding sites (Zhu et al., 2021b). However, Direct evidence establishing the regulatory role of these chromatin modifications on Dux expression is still needed. Research indicates that the activation and expression of Dux in mESCs are inhibited by epigenetic modifiers, including the H3K9 histone methyltransferases SETDB1 and G9a, as well as KAP1 and TRIM66, to maintain pluripotency (Maksakova et al., 2013; Rowe et al., 2013; De Iaco et al., 2017). Further embryo chimera studies confirm that these 2C-like cells can develop into both embryonic and extraembryonic tissues. Mechanistically, the C-terminal PDH finger domain of the chromatin reader protein TRIM66 recognizes the H3K4-K9me3 modification and suppresses Dux expression by recruiting the co-repressor DAX1 to the Dux promoter (Zuo et al., 2022). The interplay between SETDB1 and KAP1 is essential for regulating key genes such as Dux, LINE1, IAP, and ERV elements through H3K9me3 modification. The loss of these factors leads to the reactivation of repressive histone modifications on repetitive elements and regulates the reprogramming of mESCs to 2CLC states in a dose-dependent manner (Rowe et al., 2013; Wu K. et al., 2020). Histone modification factors play a pivotal role in facilitating Dux expression. The long-dispersed nuclear retrotransposon element 1 (LINE1) LINE-1 is the most abundantly expressed retrotransposon in mammals, laying a crucial role in mediating high levels of transcription at the 8-cell stage during ZGA (Rodriguez-Terrones and Torres-Padilla, 2018). Recent studies have shown that LINE-1 is reactivated at the 2-cell stage and promoting ZGA in early mouse embryos (Zhang et al., 2020). In mESCs, both ASO-mediated knockdown of LINE-1 RNA and CRISPRi-mediated silencing of LINE-1 transcription significantly downregulated of minor ZGA genes, including zscan4, Eiflad6, and OBOX4, which results in delayed development at the 2-cell stage (Li et al., 2024). Importantly, premature silencing of LINE-1 decreases chromatin accessibility at this stage, whereas embryos that sustain LINE-1 expression exhibit a more decondensed chromatin structure (Jachowicz et al., 2017). Additionally, LINE1 inhibits Dux expression by acting as a nuclear RNA scaffold that interacts with Nucleolin (NCL) and TRIM28 (KAP1). LINE1 RNA mediates the binding of NCL and KAP1 to rDNA, promoting rRNA synthesis and ESC self-renewal (Percharde et al., 2018; Sun et al., 2022). The role of KAP1 in repressing the Dux locus is well-documented, and the activation of some 2C genes occurs in its absence. LINE1 also participates in 2CLC reprogramming through the RNA modification N6-methyladenosine (m6A). This mechanism involves LINE1 RNA methylation by the m6A methyltransferase METTL3, followed by recognition of m6A by the YTH domain-containing protein 1 (YTHDC1), which recruits H3K9me3 histone methylation factors SETDB1 and KAP1 to the Dux locus, thereby suppressing Dux transcription (Figure 1) (Liu et al., 2021; Xu et al., 2021). This process not only regulates the transition from mESCs to pluripotent 2CLC but also mediates the silencing of endogenous transposon elements such as IAP, ERVK, and LINE1. Deletion of YTHDC1 and/or METTL3 results in the generation of 2C-like cells (Kasowitz et al., 2018; Chelmicki et al., 2021).

The higher-order structure of chromatin is an significant factor in epigenetic modifications and appears to play a critical role in regulating 2C genes and the transition to 2CLC (Olbrich and Ruiz, 2022). The transcription factor Dux enhances chromatin accessibility, facilitating the activation of 2CLC. Recent studies have revealed that ribosomal RNA (rRNA) suppresses the activation of 2C genes such as Dux through mechanisms involving nucleolar liquid-liquid phase separation and the higher-order structure of peri-nucleolar chromatin. It is becoming increasingly clear that inhibiting rRNA production can profoundly impact cellular fate transitions by altering nucleolar phase separation and chromatin structure, thereby releasing the inhibition of critical genes like Dux and promoting the transition of ES cells to 2CLC (Yu et al., 2021). In previous study, depletion of the RNA-binding protein LIN28, which is localized in the nucleolus, led to a significant decreased in H3K9me3 epigenetic modification of Dux and its downstream target genes, while increasing the proportion of 2C-like cells. LIN28 interacts with Nucleolin (NCL) and transcriptional silencing factor TRIM28 (KAP1). As well as with snoRNA and rRNA within the nucleolus, contributing to its structural integrity. Knockout of LIN28 reduces the binding of the NCL/TRIM28 complex to the Dux locus, thereby alleviating the inhibitory effect on Dux gene expression and inhibiting rRNA transcription (Sun et al., 2022). However, the exact mechanism by which LIN28 regulates H3K9me3 modification at the peri-nucleolar chromatin remains unclear.

In addition to histone modifications, various epigenetic mechanisms are involved in activating Dux expression to regulate the reprogramming of ESCs into the 2CLC state. During pre-implantation embryo development, bioinformatics analyses have shown a general decrease in DNA methylation levels (Wang et al., 2018). Specifically, at the 2-cell stage, the Dux promoter region undergoes demethylation, suggesting that the initial activation of Dux depends on DNA demethylation (Ruebel et al., 2019). Cytosine methylation on CpG islands of gene regulatory elements such as promoters, spatially blocks the binding of transcription factor complexes to DNA.

Previous studies have indicated that in early mouse embryos, the 5 mC oxidation enzyme TET protein forms a complex with the chromatin architectural protein SMCHD1, which negatively regulates TET protein activity. Consequently, loss of SMCHD1 in mouse embryonic stem cells leads to DNA hypomethylation and demethylation of the Dux promoter, thus activating Dux expression and facilitating the reprogramming of the early embryo genome to the 2CLC state (Huang et al., 2021). Furthermore, knockdown of SMCHD1 via siRNA in fertilized eggs results in sustained overexpression of Dux from the 2-cell to the 8-cell stage (Ruebel et al., 2019). Additionally, TET appears to have a dual role in regulating the transition to 2C-like cells, depending on its interacting proteins. TET-mediated DNA demethylation also influences site-specific high methylation and the downregulation of 2C-specific genes, including Dux, through the knockout of maternal factors GADD45a/b/g (Schule et al., 2019). Maternal factors are crucial during the ZGA phase of early development (Li et al., 2010; Ancelin et al., 2016; Wasson et al., 2016). Maternal factor 3 (Dppa3) promotes DNA demethylation in ESCs and reshapes chromatin accessibility at 2C-specific genes like Zscan4 and MERVL elements, thereby promoting the expression of 2C-like genes and the transformation of 2CLC (Zhang et al., 2022). However, RNA-seq results indicate that while Dppa3 activates the expression of 2C-like genes such as Tcstv1/3, Zscan4, and MERVL, it does not affect Dux expression (Mazid et al., 2022). It suggests that Dppa3’s crucial function occurs post-fertilization and before the 2-cell stage.

Interestingly, highly expressed genes such as Dux and Zscan4 exhibit significantly elevated H3K4me3 deposition in their promoter regions. (Zhang et al., 2021). Recent findings also highlight the prevalent presence of H3K27ac modifications during the late 2-cell stage of zygote genome activation (ZGA). Many broad H3K27ac domains overlap with H3K4me3 domains (Wu et al., 2023). Both H3K4me3 at promoter regions and H3K27ac at enhancer regions are associated with histone activity. The complex interplay between chromatin remodeling proteins underscores the intricate nature of this regulatory network, providing insights into the mechanisms governing pluripotency and cell fate determination.

### Transcription factor proteins modulate the activation of Dux to govern the fate transition of 2CLC

The discussion also examines the role of the transcription factor Dux in driving the transition of mouse embryonic stem cells (mESCs) into and out of a transient 2-cell-like pluripotent state (2CLC). This process involves not only epigenetic modifications but also maternal upstream regulators that influence cellular fate transformation. However, the comprehensive regulation of cell fate remodeling by these factors remains to be fully elucidated.

Recent studies have illuminated the complex interplay between Dux and other regulatory factors, such as Dppa2, Dppa4, and NELFA. These pluripotency factors and maternal regulators directly impact Dux expression by binding to its promoter regions and promoting its transcription. The heterogeneous expression pattern of NELFA in mESCs adds further complexity, with NELFA overexpression leading to increased binding at Dux loci. This upregulation of NELFA is associated with the enhanced expression of key 2-cell genes, including Zscan4 and the endogenous retrovirus MERVL, underscoring NELFA’s crucial role in orchestrating cellular fate. Notably, a significant proportion of MERVL + cells exhibit high levels of NELFA expression. Mechanistic studies have demonstrated that NELFA, operating independently of the NELF complex, interacts with DNA topoisomerase II alpha (Top2a) to bind Dux sites and activate its transcription, thereby facilitating the transition to a 2CLC state (Figure 1) (Hu et al., 2020).

Similarly, Dppa2 and Dppa4 have been identified as positive regulators in the induction of 2CLC and activation of zygote genome activation (ZGA) genes. Depletion of both Dppa2 and Dppa4 in embryonic stem cells impairs reprogramming into 2CLC but does not affect pluripotency or self-renewal capabilities. Overexpression of Dux can rescue the 2CLC population in Dppa2/Dppa4 double knockout cells, although it does not reverse the knockout phenotype (De Iaco et al., 2019; Chen et al., 2021). During induced pluripotent stem cell (iPSC) reprogramming, Dppa2 and Dppa4 exhibit transient upregulation during the 2C-like conversion phase. Mechanistically, these factors bind to both the promoter and gene body of Dux, driving its upregulation and promoting the 2C-like conversion program (Eckersley-Maslin et al., 2019).

Post-translational modification by SUMO (Small Ubiquitin-like Modifier) has been identified as a critical inhibitory factor in regulating the fate of 2CLC, influencing both somatic and pluripotent states through targeted modifications of various substrates (Cossec et al., 2018). In ESCs, SUMOylation mediates the entry into and exit from the 2CLC state through at least two distinct mechanisms: Firstly, SUMOylation modifications are involved in maintaining H3K9me3 levels across the genome, including Dux sites, This modification recruits inhibitory complexes such as KAP1 or SETDB1 to these regions, suppressing Dux gene expression, and thereby preventing chromatin opening and the transition to a pluripotent state (Theurillat et al., 2020). Secondly, SUMOylation regulates pluripotency-regulating factors, The subunit of the non-canonical PRC1 complex (PRC1.6), Rif1, is recruited directly to Dux sites and is also regulated by SUMOylation (Li et al., 2022). Additionally, the SUMO E3 ligase PIAS4 mediates the SUMOylation of Dppa2, leading to its inactivation and consequently hindering the transition to a 2CLC state (Yan et al., 2019). These findings underscore the intricate regulatory network involved in orchestrating cellular fate transitions and highlight the multifaceted roles of various transcription factors and regulatory proteins.

### The direct regulation of MERVL activation is a key determinant in the reprogramming of 2CLC

The transcription factor Dux is critical for the fate transition of embryonic stem cells (ESCs), as its expression is sufficient to induce 2-cell-like cells (2CLCs) with transcriptional and epigenetic features akin to those of 2-cell embryos. This includes the activation of hallmark 2CLC elements such as the MERVL retrotransposon and 2C-specific transcripts. However, research on DUX knockout mice has shown that while DUX is important for development, it is not absolutely essential (Guo et al., 2019). This finding suggests that there are parallel and redundant mechanisms *in vivo* capable of activating MERVL and regulating zygote genome activation (ZGA), thereby maintaining the pluripotent state of cells (Chen and Zhang, 2019). Notably, only approximately 25% of the chromatin regions accessible in 2CLC are bound by Dux, indicating the presence of compensatory mechanisms *in vivo* that may drive and sustain 2CLC reprogramming (Bosnakovski et al., 2021). Furthermore, studies have demonstrated that the activation of MERVL repeat sequences alone is sufficient to increase the proportion of 2CLC in culture, highlighting the crucial role of MERVL transcription in early embryonic development.

### Atypical histone modifications regulate the activation of MERVL and control the fate transformation of 2CLC

The chromatin remodeling factor CAF-1 and its subunits play a crucial role in inhibiting the activation of endogenous retrovirus MERVL in ESCs (Yankulov, 2015). Research indicates that CAF-1 is involved in establishing modifications on the non-canonical histone H3.3, which are essential for silencing endogenous retroviral elements within ESCs (Elsasser et al., 2015). Chromatin immunoprecipitation followed by sequencing (ChIP-seq) data reveal the enrichment of H3.3 at the Dux locus, where it acts as a critical upstream regulator by suppressing Dux activation (Tian et al., 2020). The deletion of CAF-1 subunits p150 or p60 in ESCs results in increased accessibility of MERVL, leading to upregulation of adjacent genes and a higher abundance of 2-cell-like cells (Yankulov, 2015). Notably, the recruitment of the H3.3 partner complex DAXX, along with H3.3 and KAP1, to endogenous retroviruses (ERVs) suggests a unique heterochromatic state where H3.3 and H3K9me3 coexist, contributing to the silencing of ERVs in ESCs.

**FIGURE 2:**
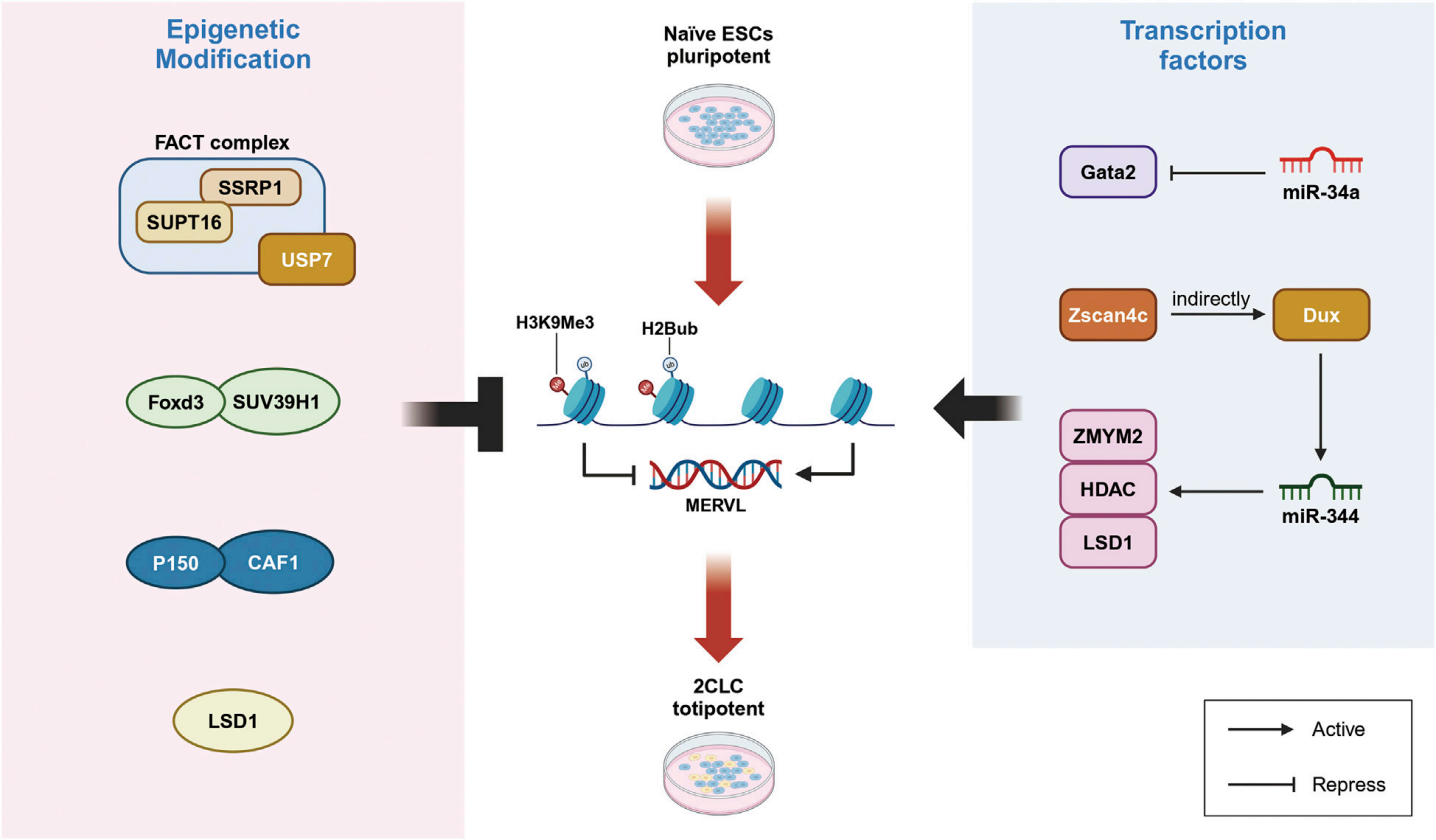
Direct regulation of MERVL activation is crucial for controlling 2-cell-like cell. Left: Atypical histone modifications play a key role in regulating MERVL activation and controlling the fate transition to 2CLC. The H2A/B histone chaperone FACT complex represses MERVL activation, while CAF-1 and its subunit p150 inhibit MERVL expression. FOXD3 recruits SUV39H1 to establish heterochromatic modifications, thereby suppressing MERVL transcription. Right: Non-coding regulatory factors are involved in the regulation of MERVL activation and the control of 2CLC fate transformation. miR-34a targets the transcription factor Gata2 to inhibit MERVL activation. Additionally, miR-344 inhibits ZMYM2 and LSD1, which relieves the repression of MERVL. The zinc finger domain of Zscan4 binds to MERVL, thereby activating gene expression.

Foxd3, a critical regulatory factor in the ES cell genome, binds to and recruits the histone methyltransferase SUV39H1 to major satellite repeat sequences and MERVL loci, establishing H3K9me3 heterochromatic modifications that suppress the transcription of repetitive elements. The loss of Foxd3 leads to de-repression of MERVL and major satellite repeat sequence, resulting in an altered equilibrium between stem cell and 2-cell-like cell populations (Puri et al., 2021). However, it remains unclear whether Foxd3 directly interacts with other regulatory factors associated with 2-cell-like cells, such as Dux, to mediate H3K9me3 modifications. In addition to histone H3 modifications, the de-ubiquitination of histone H2B, specifically H2Bub, also plays a role in inhibiting MERVL expression. In mouse ESCs, the loss of the H2A/H2B histone chaperone FACT component Ssrp1 results in the activation of MERVL, MERVL and an increase in the proportion of 2-cell-like cells. Further investigations have shown that Ssrp1 interacts with the H2B de-ubiquitinates Usp7, recruiting it to the MERVL loci to suppress its expression. Inhibition of Usp7 replicates the phenotype observed with Ssrp1 deletion (Figure 2) (Chen et al., 2020).

### Non-coding regulatory elements play a role in modulating MERVL activation, which in turn controls the fate transformation of 2CLC

MicroRNAs (miRNAs) are small non-coding RNAs that play crucial roles in post-transcriptional gene regulation through mechanisms such as mRNA degradation and translation inhibition. These molecules are essential for normal developmental processes and the maintenance of pluripotent stem cell identities (Judson et al., 2009). In both embryonic stem cells (ESCs) and induced pluripotent stem cells (iPSCs), the absence of miR-34a results in the upregulation of endogenous retroviruses, such as MERVL, facilitating the emergence of a totipotent 2-cell-like cell (2CLC). Mechanistically, miR-34a inhibits MERVL activation by targeting the transcription factor Gata2. Therefore, Gata2 knockdown and miR-34a overexpression both negatively impact 2CLC reprogramming (Choi Y. J. et al., 2017).

Intriguingly, miR-34a targets multiple factors that collaboratively restrict cell fate potential and suppress MERVL expression in pluripotent stem cells. Similarly, miR-344 has been shown to facilitate 2CLC reprogramming. The activation of endogenous MERVL or the overexpression of miR-344 alone is sufficient to induce 2C-specific gene expression, driving ESC reprogramming toward 2CLC (Yang et al., 2020). ZMYM2 recruit of an LSD1/HDAC co-repressor complex to MERVL, thereby inhibiting LTR-driven transcription. miR-344 functions post-transcriptionally to inhibit ZMYM2 and its partner LSD1, thus relieving the suppression of MERVL-LTR (Figure 2). Consistent with this, zygotic deletion of ZMYM2 results in impaired LSD1 targeting of MERVL and 2C-specific genes, while miR-344-mediated repression of LSD1 leads to MERVL activation. Notably, Dux interacts with and activates the transcription of miR-344 (Guzzo et al., 2014). Furthermore, the zinc finger domains of ZMYM2 contain Sumoylation sites, which, when modified by PIAS4, lead to the suppression of MERVL-LTR-driven transcription.

It is noteworthy that Zscan4c-mediated activation of MERVL and subsequent expression of 2C-specific genes are crucial for acquiring 2-cell-like features during cellular reprogramming. The zinc finger domain of Zscan4c directly binds to the LTR region MT2 of MERVL, enhancing MT2’s activity as an enhancer and initiating the expression of MERVL genes. This activation also influences the expression of adjacent 2C/4C genes. Additionally, MERVL activation is associated with increased histone modifications, specifically H3K27ac and H3K14ac, on the MT2 domain (Zhang W. et al., 2019). Research indicates an intermediate population characterized by elevated Zscan4 transcript levels during the transition from ESCs to 2CLCs, suggesting that these cells are precursors to 2-cell-like cells (Rodriguez-Terrones et al., 2018). This highlights the critical role of the Zscan4 gene in activating 2C-specific genes and underscores its importance in 2CLC reprogramming.

Studies have shown that the pluripotency regulator Zfp281 primarily inhibits the upregulation of transcripts during 2CLC conversion by targeting Tet1, thus impeding 2CLC reprogramming. Notably, Zfp281 does not directly interact with MERVL. Its knockdown does not affect the expression of reporter genes controlled by the MERVL promoter or Dux, but rather facilitates the activation of Zscan4d due to RNA splicing inhibition (Wen et al., 2022). Conversely, the transcription factor Myc regulates a cascade of events that involves the downregulation of pluripotency genes and the subsequent inhibition of 2C-specific gene activation via Dnmt1 (Fu et al., 2019). The high expression of the endogenous retrovirus MERVL in 2-cell cleavage-stage embryos suggests that MERVL plays a role beyond being a marker of the 2C-like transcriptional and epigenetic state. The MERVL-driven rewiring of gene regulatory networks that induces numerous 2C-specific genes may contribute to the establishment and maintenance of the functional fate potential of 2C-like cells. Further research is needed to elucidate the detailed mechanisms underlying these processes and their implications for embryonic development and cell fate determination.

### Small molecule compounds induce the fate transformation of 2CLC

Currently, the generation of 2CLCs *in vitro* largely relies on gene editing techniques targeting epigenetic modifiers and transcription factors. However, ethical concerns associated with these methods have hindered the broader application of 2CLCs in regenerative medicine. To address these challenges, some research groups are now concentrating on screening and identifying small molecules that can induce the formation of 2CLCs without the need for gene editing. These small molecules also hold potential for supporting the *in vitro* cultivation of early embryos. These innovative approaches provide promising avenues for advancing regenerative medicine while navigating the complex ethical landscape.

ESCs cultured in Serum + LIF (S/L) medium retain their metastable pluripotency and self-renewal capacity (Kalkan et al., 2019). In contrast, ESCs cultured in 2i + LIF (2iL) medium, which includes inhibitors of the MEK and GSK3 pathways, exhibit characteristics closely resembling those of the inner cell mass of pre-implantation embryos, but with enhanced differentiation potential (Choi J. et al., 2017). Despite these distinct culture conditions, a small fraction of ESCs (approximately 0.1–1%) still transition into 2-cell-like cells (2CLCs) under both conditions. This finding highlights the significant role that different culture media play in maintaining stem cell properties, yet the specific factors driving the activation of 2C-like cells remain to be fully elucidated.

Studies have reported that activation of the retinoic acid (RA) signaling pathway increases the proportion of 2C-like cell subpopulation within ESCs cultured in Serum + LIF (S/L) medium (Wu B. et al., 2020; Iturbide et al., 2022). RA promotes the transition of ESCs to a 2C-like state by enhancing the expression of Dux and Duxbl1, or by activating the PRAME family member Gm12794c (Tagliaferri et al., 2019; Napolitano et al., 2020). Additionally, RA treatment is associated with decreased DNA methylation, increased histone modifications, reduced glycolytic activity, and lower protein synthesis levels in ESCs. Beyond its role as an upstream regulator of the 2C-like state in chemically defined culture conditions, RA has also been implicated in driving the NELFA-mediated 2C-like state in mouse ESCs(Wang et al., 2021).

**FIGURE 3:**
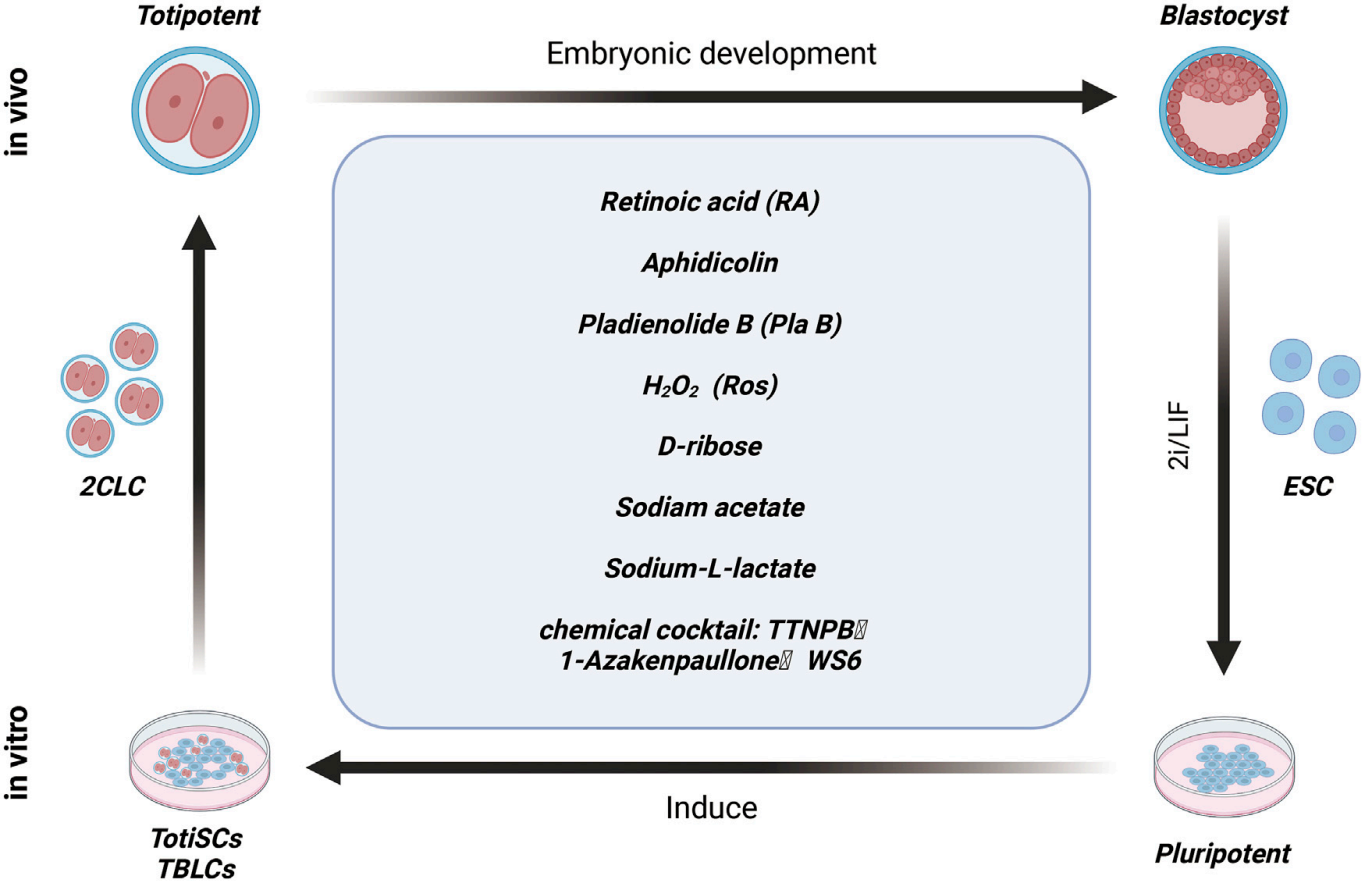
Small molecule compounds induce the fate transformation of 2CLC.

In addition, with these research findings, an increasing number of small molecule compounds have been identified as inducers of 2CLC fate transformation. Notable examples include L-lactate, D-ribose, and sodium acetate, each of which induce 2CLC conversion in a dose-dependent manner, with sodium acetate demonstrating a particularly strong inductive effect (Rodriguez-Terrones et al., 2020). Recent studies have also highlighted the role of aphidicolin (APH), a reversible DNA polymerase inhibitor, in triggering the formation of a subset of 2CLC cells. APH activates ATR (ataxia telangiectasia and Rad3-related protein), which promotes the binding of the GSRF1 protein to Dux mRNA, thereby driving Dux activation and facilitating 2CLC reprogramming (Figure 3) (Atashpaz et al., 2020). The study underscores the potential of small molecular in modulating cellular pathways related to 2CLC fate determination.

Over the years, maintaining induced pluripotent stem cells (iPSCs) *in vitro* for extended periods has posed a significant challenge in stem cell research. Recent efforts have involved screening thousands of small molecule combinations, leading to the identification of the TAW cocktail—comprising TTNPB, 1-Azakenpaullone, and WS6—as effective in inducing mouse pluripotent stem cells (PSCs) to convert into chemically induced totipotent stem cells (ciTotiSCs) (Figure 3). This cocktail enables the long-term stable culture of these cells *in vitro*. Characterization of the induced ciTotiSCs has shown that they exhibit similarities to 2-cell embryos at the transcriptional, epigenomic, and metabolomic levels, as well as demonstrating bidirectional developmental potential (Hu et al., 2023). Additionally, a previous study reported that introducing the splicing inhibitor PLAB to Serum + LIF (SL) culture conditions allows embryonic stem cells (ESCs) to be reprogrammed into totipotent blastocyst-like cells (TBLCs), which can be stably cultured *in vitro*. Further analysis indicated that TBLCs share molecular characteristics and developmental potential with 2-cell and 4-cell blastocyst embryos (Shen et al., 2021). However, it remains uncertain whether these culture conditions are applicable for the reprogramming of human ESCs.

These findings highlight the complex metabolic dynamics involved in early embryonic development and the maintenance of pluripotency. Prior to implantation, pre-implantation mouse embryos depend exclusively on monocarboxylates, such as acetate and lactate, to fulfill their bioenergetic demands until the 8-cell stage (Houghton et al., 1996). In contrasts, embryos at the morula and blastocyst stages shift to using glucose, which supports energy production through a combination of glycolysis and oxidative phosphorylation (Leese and Barton, 1984). Research has identified an abnormal redox state in spontaneously generated 2-cell-like cells (2CLCs) in ESC cultures, marked by elevated levels of reactive oxygen species (ROS) compared to normal ESCs. Additionally, Treatment with H2O2 has been shown to activate the conversion program in mESCs, resulting in an increased population of 2CLCs (Zhang C. et al., 2019). This aberrant redox state suggests that targeting redox signaling pathway may present novel strategies for improving the efficiency and accuracy of induced cell fate transitions.

## Conclusion and future perspectives

2CLCs reprogrammed from ESCs exhibit characteristics of pluripotency and possess a broader developmental potential. Current investigations underscore the complexity of a regulatory network that encompasses protein transcription factors, non-coding RNAs, and small molecules, collectively orchestrating the transition of ESCs to 2CLCs (Iturbide and Torres-Padilla, 2020). The detailed molecular mechanisms underlying these regulatory pathways have also been elucidated. Despite these advancements, existing pluripotent cell models are predominantly developed *in vitro*, which limits our understanding of pluripotency during oogenesis and pre-implantation development (Ren et al., 2022). *In vitro*, most 2CLCs tend to spontaneously revert to the ESC state, and the underlying mechanisms governing this transition are not well-understood. Recent studies have revealed that during the conversion from 2CLCs back to ESCs, pluripotency genes are activated in a biphasic manner, with the degradation of Dux mRNA being a crucial factor in the exit from the 2C-like state. This process is distinct from the spontaneous entry into the 2C-like state.

Single-cell RNA sequencing (scRNA-seq) data have been employed to elucidate the dynamic alterations in the transcriptional landscape that occur during the transition of 2CLCs from the 2C state. This analysis revealed distinct intermediate states characterized by differential expression of endogenous retrovirus (ERV) genes throughout the transition from the 2C state. Each state was associated with unique cell cycle phase preferences, with 2CLCs showing enrichment in the G1 and G2/M phases compared to the S phase. Notably, the inhibition of ESCs at specific cell cycle stages, notably in the G1/early S phase, was sufficient to activate ERVs and initiate the reprogramming process towards 2CLCs. These findings suggest that both the entry into and exit from the 2CLC state during *in vitro* culture are regulated by a multifaceted regulatory framework rather than a singular pathway, implying the engagement of upstream regulatory elements.

Despite considerable progress in understanding the pathways and molecular mechanisms governing the reprogramming of 2CLCs, a number of unresolved questions persist. For instance, do additional epigenetic modifiers and transcriptional regulators participate in the induction of ESC fate conversion? How do the regulatory relationships between various epigenetic modifications during the reprogramming process of 2CLCs? Further investigations are needed to address these questions and to fully elucidate the complex regulatory networks involved in 2CLC reprogramming.

The potential impact of actively transcribed LTR elements on genomic stability during early embryonic development is a crucial area for investigation. Understanding the mechanisms underlying the epigenetic silencing and activation of LTR elements during 2-cell-like cell (2CLC) reprogramming and elucidating how these mechanisms maintain the delicate balance between activation and repression, poses a significant challenge and warrants focused research.

In summary, the *in vitro* induction of pluripotent stem cells provides a valuable model for exploring the origin of life. The generation of ES cell lines and cloned animals through somatic cell nuclear transfer has demonstrated that the cytoplasm of an oocyte can reprogram a somatic cell’s genome to an embryonic state (Aboul-Soud et al., 2021). However, recent studies have shown that the fusion of somatic cells, such as fibroblasts or T lymphocytes, with human embryonic stem cells (ESCs) can also reprogram the somatic cell nucleus to a pluripotent state (Cowan et al., 2005). This advancement highlights the potential of using ESCs as an alternative to oocytes for reprogramming human somatic cell nuclei, paving the way for the creation of clinically relevant cell types for transplantation. Furthermore, embryonic stem cells provide an alternative source for growing organoid tissues, which is especially important for tissues lacking established protocols for organoids derivation from adult stem cells, such as the brain, kidney, inner ear, retina, and thyroid (Nuciforo and Heim, 2021). In summary, ESCs are utilized to derive various specific cell types based on their pluripotency, and cell-based gene therapies hold significant potential in the treatment of cancer and regenerative medicine. Applying this knowledge to enhance SCNT and treat developmental disorder diseases will be a critical step in translating these findings into clinical practice.

With advancements in single-cell sequencing technologies and improvements in bioinformatics tools, future studies are expected to unveil the intricate regulatory networks that govern pluripotent cell reprogramming. By leveraging cutting-edge technologies and interdisciplinary approaches, researchers are likely to gain deeper insights into the complex processes of cellular plasticity and differentiation. This understanding could significantly advance the fields of developmental biology and regenerative medicine. Nonetheless, substantial progress is still needed to achieve precise control the production of pluripotent cells and translate these findings into practical medical applications using targeted activators and inhibitors.
