# Corrigendum: Advances in understanding the regulation of pluripotency fate transition in embryonic stem cells

**DOI:** 10.3389/fcell.2024.1523967

**Published:** 2024-11-15

**Authors:** Yong kang Jia, Yang Yu, Li Guan

**Affiliations:** ^1^ School of Life and Health Sciences, Hubei University of Technology, Wuhan, China; ^2^ Guangzhou Women and Children’s Medical Center, Guangzhou Medical University, Guangzhou, China

**Keywords:** ESCs, 2-cell-like cells, Dux, MERVL, epigenetic modification, cell fate reprogramming

In the published article, there was an error in **Affiliation 1**. Instead of “^1^School of Life and Health Sciences, Huei University of Technology, Wuhan, China,” it should be “^1^School of Life and Health Sciences, Hubei University of Technology, Wuhan, China.”

The authors apologize for this error and state that this does not change the scientific conclusions of the article in any way. The original article has been updated.

